# Effects of microplastic release from 3D-printed orthodontic aligners: a histological and immunological bioassay approach

**DOI:** 10.1093/ejo/cjag001

**Published:** 2026-05-27

**Authors:** Marina Borgese, Piero Antonio Zecca, Annalisa Grimaldi, Nicolò Baranzini, Mario Raspanti, Francesca Zara, Marco Serafin, Alberto Caprioglio

**Affiliations:** Department of Medicine and Technological Innovation, University of Insubria, Via Guicciardini 9, Varese 21100, Italy; Department of Medicine and Technological Innovation, University of Insubria, Via Guicciardini 9, Varese 21100, Italy; Department of Biotechnology and Health Sciences, University of Insubria, via J.H. Dunant 3, Varese 21100, Italy; Department of Biotechnology and Health Sciences, University of Insubria, via J.H. Dunant 3, Varese 21100, Italy; Department of Medicine and Technological Innovation, University of Insubria, Via Guicciardini 9, Varese 21100, Italy; Department of Biomedical, Surgical and Dental Sciences, University of Milan, via della Commenda 10, Milan 20122, Italy; Department of Biomedical, Surgical and Dental Sciences, University of Milan, via della Commenda 10, Milan 20122, Italy; Department of Biomedical, Surgical and Dental Sciences, University of Milan, via della Commenda 10, Milan 20122, Italy; Fondazione IRCCS Cà Granda, Ospedale Maggiore Policlinico, via della Francesco Sforza 35, Milan 20122, Italy

**Keywords:** 3D-printed aligners, biocompatibility, immune response, micro-nanoplastics, oxidative stress

## Abstract

**Aim:**

This study investigated the acute biological effects of micro- and nanoplastics (MPs/NPs) released from 3D-printed orthodontic aligners, focusing on their potential to elicit inflammatory and oxidative responses. Given the dynamic intraoral environment, the use of a biologically relevant invertebrate model (*Hirudo verbana*) enabled histological and molecular assessment of short-term exposure to plastic-derived particles.

**Materials and methods:**

MPs/NPs were generated from 3D-printed aligner samples via mechanical stress and ultrasonication. Medicinal leeches were exposed to the resulting plastic dispersion for 24, 72 h, and 1 week (triplicate experiments). Morphological alterations were assessed by light and electron microscopy. CD31 and HmAIF-1 immunostaining and acid phosphatase assays evaluated immunological responses. Oxidative stress was quantified by quantitative PCR (qPCR) analysis of *SOD* and *GST* gene expression. One-way ANOVA with Dunnett’s post hoc test comparing MP/NP-treated vs controls (*α* = 0.05).

**Results:**

Exposure to MPs/NPs triggered rapid angiogenesis, confirmed by increased CD31 expression and vascular remodelling over time (24 h, *P* < .01; 72 h, *P* < .001; 1 week, *P* < .01). Macrophage-like cell activation was significantly evidenced by elevated *Hm*AIF-1 levels (24 h, *P* < .001; 72 h, *P* < .01; 1-week, *P* < .001) and acid phosphatase activity (24 h, *P* < .001; 72 h, *P* < .01; 1-week, *P* < .001). Oxidative-stress markers (*SOD* and *GST*) were significantly upregulated at 24 h (*P* < .001) and, for glutathione transferase only, slightly increased at 72 h (*P* < .05), indicating an acute cellular stress response that decreased at later time points.

**Conclusions:**

Even brief exposure to MPs/NPs released from 3D-printed aligners can disrupt tissue homeostasis, activating inflammatory and oxidative pathways. These findings raise concerns about the biocompatibility of photopolymer resins and underscore the need for more dynamic, biologically relevant testing models to evaluate the safety of orthodontic materials under clinically realistic conditions.

## Introduction

Aligners have revolutionized conventional orthodontic treatment, providing patients with a nearly invisible, removable, and highly customized alternative to traditional braces. Unlike fixed appliances, aligners are thin trays that fit over teeth and progressively shift them into the desired alignment. These devices were conventionally manufactured through thermoforming [1]. However, advancements in material science have introduced 3D printing as a novel production method by using photopolymerizable resins [2, 3].

Despite their advantages, concerns remain about the biocompatibility of light-cured resins, in particular their release of microplastics (MPs) and nanoplastics (NPs) [4–6]. These pollutants, increasingly recognized for their health risks [7], are defined as plastic fragments <5 mm (MPs) and <1 µm (NPs), with physicochemical properties enabling them to penetrate biological membranes [8, 9].

During orthodontic treatment, aligners are exposed to constant mechanical, thermal, and chemical stress. Functional and parafunctional activities, along with temperature fluctuations and salivary enzymes, promote material degradation and polymer breakdown, leading to the release of MPs and NPs into the oral cavity [6, 10–12]. These particles may be ingested, absorbed by the oral mucosa, or inhaled, raising concerns about potential systemic effects upon entering the human bloodstream [13, 14].

While *in vitro* studies have assessed the cytotoxicity of MPs and NPs in mammalian cells, their long-term effects during orthodontic aligner wear remain unclear [15]. Given the prolonged exposure of oral tissues, it is essential to evaluate their histological and molecular impact under dynamic conditions. Although mammalian models could provide insights, ethical concerns limit their use [16]. The medicinal leech represents an alternative bioassay model to assess the biological effects of plastics from 3D-printed aligners.

*Hirudo verbana* is a well-established invertebrate model in toxicological studies due to its simple anatomy, functional circulatory system, and botryoidal tissue involved in haematopoiesis and immune responses [17–19]. Its permeable epithelium facilitates nanoparticle absorption, and its immune and inflammatory responses closely resemble those of higher organisms, making it a suitable bioassay model for evaluating the effects of MPs and NPs [20]. Moreover, *H. verbana* provides a simple and accessible model for rapid toxicity assessment, featuring conserved innate immune and regenerative pathways homologous to those of vertebrates. Its mucosal surface mimics the humid oral environment, making it suitable for evaluating plastic exposure. Prior studies have shown that MPs trigger inflammatory and oxidative-stress responses in this model [21].

Therefore, the objective of this study was to conduct a comprehensive histological and molecular analysis of the effects of MPs and NPs released from 3D-printed aligners, using medical leeches as experimental model organisms. By simulating interactions between these particles and mucosal tissues, we aimed to assess their cytotoxicity, inflammatory responses, and broader implications for oral and systemic health.

## Materials and methods

### Sample manufacturing and microplastics/nanoplastics generation

Samples of size 5 × 5 × 0.5 mm^3^ were fabricated using a commercially available 3D-printable photopolymer resin; its chemical composition remained undisclosed due to patent protection, despite previous investigations identifying the presence of both polyurethane and acrylate compounds, based on urethane acrylate oligomers (aliphatic vinyl-urethane monomers with acrylate and/or methacrylate functionality) [22]. Before testing, specimens were placed in sterile Petri dishes pretreated with piranha solution to remove organic residues and ensure a contaminant-free surface. The samples were then subjected to a UV sterilization cycle.

To simulate the occlusal stress, plastic specimens were immersed in ultrapure water and mechanically abraded under controlled conditions. Following mechanical abrasion, samples and water were subjected to ultrasonication at 37°C for 15 min to facilitate the detachment of MPs and NPs into solution [23]. The final dispersion had an estimated concentration of 0.005 mg/ml of MPs/NPs, as quantified in a previous study on MP/NP particle generation [4].

### Animal sample preparation

Medicinal leeches (*H. verbana*, *Annelida, Hirudinea*) (Italian Leech Farm ILFARM S.r.l., Varese, Italy) were kept in 5 ml of salted water (NaCl 1.5 g/L) at a constant temperature of 20°C. Animals were randomly divided into three experimental groups (24, 72 h, and 1 week timepoints) and exposed to an MPs/NPs solution.

After 24, 72 h, and 1 week, leeches were anaesthetized by a 10% ethanol solution until they appeared unresponsive and then sacrificed. All experiments were performed in triplicate, and leeches not exposed to MPs/NPs (nontreated, N.T.) served as the control group; each group consisted of five leeches.

All animal experiments in this study were conducted in strict accordance with the ARRIVE guidelines [24], the U.K. Animals Act and its associated guidelines [25], as well as the EU Directive 2010/63/EU on the protection of animals used for scientific purposes [26]; the use of *H. verbana* is not among the animals protected under that directive.

### Morphological analysis at light and transmission electron microscopy

All sacrificed samples were fixed in 4% glutaraldehyde diluted in 0.1 M cacodylate buffer (pH 7.4) overnight, then reduced to smaller fragments to optimize the inclusion step. Subsequently, after three consecutive washes with buffer, the samples were placed in 2% osmium tetroxide (OsO4) for 1 h at room temperature, and then dehydrated using an increasing ethanol series (50%, 70%, 90%, uranyl acetate, and 100%). Once dehydration was complete, the leech’s tissues were transferred to propylene oxide for 30 min and then immersed in a 1:1 solution of resin and propylene oxide for 30 min. Subsequently, they were transferred to a 1:2 solution of propylene oxide and resin, left overnight, and then immersed in Epon-Araldite 812 epoxy resin (Sigma-Aldrich, Milan, Italy). The resin was ultimately cured at 70°C overnight.

Sections for light microscopy (0.7 μm in thickness) were then obtained using a RMC Power Tome XL (Boeckeler Instruments, USA) ultramicrotome and stained with crystal violet (1 g/100 ml) and basic fuchsin (0.13 g/100 ml). Samples were observed under the Eclipse E600 optical microscope (Nikon Instruments, Inc., Melville, NY, USA), and images were captured with the DS-5M-L1 digital camera (Nikon). Instead, ultrathin sections (0.07 μm in thickness) were placed on 300-mesh copper grids (Sigma-Aldrich, Milan, Italy), counterstained with uranyl acetate and lead citrate, and analysed with a JEOL1400Plus transmission electron microscope (JEOL, Tokyo, Japan). Data were recorded using a MORADA digital camera system (Olympus, Tokyo, Japan).

### Immunological and acid phosphatase analyses

For immunological and acid phosphatase (ACP) assays, dissected leeches were immediately included in optimal cutting temperature (OCT) (Tissue-Tek OCT compound, Sakura Finetek, Torrance, CA, USA) and then gradually frozen in dry ice/liquid nitrogen. Cryosections (0.7 µm) were obtained using a CM1850 cryostat (Leica Biosystems, Nussloch, Germany), stored at −20°C, and then treated differently for the following techniques.

Cryosections were rehydrated for 10 min in phosphate-buffered saline (PBS) and subsequently incubated in a 2% bovine serum albumin (BSA) blocking solution containing 0.1% Tween (Sigma-Aldrich) for 30 min, which was also used for antibody dilution.

For immunological assays, samples were incubated for 1 h at room temperature with the following primary antibodies: mouse monoclonal anti-CD31 primary antibody (Novocastra Laboratories Ltd, Nussloch, Germany) and rabbit polyclonal anti-*Hm*AIF-1, diluted 1:200 and 1:500, respectively. Subsequently, the slides were subjected to three consecutive washes in PBS buffer and then incubated with antimouse and antirabbit fluorescein isothiocyanate-conjugated secondary antibodies (FITC; Thermo Fisher Scientific, Inc., Waltham, MA, USA), diluted 1:300, for 45 min at 20°C. After three washes in PBS buffer, cell nuclei were counterstained with 0.1 mg/ml DAPI (4,6-diamidino-2-phenylindole), diluted in PBS for 6 min, and the slides were mounted with a PBS/Glycerol Citifluor solution (Citifluor Ltd., London, UK). Primary antibodies were omitted in negative control experiments.

Samples were analysed using a DS-SM fluorescence microscope (Nikon), and staining was visualized with excitation/emission filters of 490/525 nm for FITC and 340/488 nm for DAPI. Acquired images were then processed and combined.

For the ACP assay, cryosections were rehydrated in PBS for 10 min and then incubated for 5 min in 0.1 M acetic acid/sodium acetate buffer. Subsequently, samples were immersed in a reaction mixture consisting of 0.1 M acetic acid/sodium acetate buffer, 0.01% naphthol phosphate, 2% N,N-dimethylformamide, 0.06% Fast Red, and 0.5 mM MnCl₂ for 90 min at 37°C. After repeated washes, slides were mounted with Citifluor and analysed under a light microscope.

### RNA extraction and quantitative PCR

Dissected tissues have been frozen in liquid nitrogen and ground. The obtained samples were then resuspended in 1 ml TRIzol (Thermo Fisher Scientific, Inc.) and centrifuged at 12 000rpm for 10 min at 4°C. Afterwards, the aqueous phase was withdrawn and incubated for 5 min at 20°C. Then, 200 μl of cold chloroform was added, and the samples were centrifuged for 15 min at 13 000 rpm at 4°C. After phase separation, 500 μl of the aqueous phase, containing the nucleic acids, was transferred to an Eppendorf tube and mixed with 500 μl of isopropanol. Samples were inverted six times, incubated on ice for 15 min, and then centrifuged at 13 000 rpm for 15 min at 4°C. Once the pellet formed, the supernatant was removed, and the samples were washed with 75% ethanol. They were centrifuged at 10 000 rpm for 10 min at 4°C, and the pellets were allowed to dry in air for 7 min before being resuspended in diethylpyrocarbonate-treated water. The extracted RNA was quantified, and its purity was assessed using a 1% agarose gel. Subsequently, 2μl of RNA was reverse-transcribed into cDNA using M-MLVreverse transcriptase (ThermoFisher Scientific, Inc.). qPCR was performed in triplicate and analysed using a CFXreal-time polymerase chain reaction (PCR) detection system (Bio-Rad Laboratories S.r.l., Segrate, Italy).

The following primers were used for the qPCR amplification of the selected genes: for the macrophage-marker *Hm*AIF-1, forward primer was 5′-GACCTCAAAGACAAGCAGGG-3′ and reverse primer was 5′-GGCCAATCTTCTCCAGCATC-3′, generating a product of 229 bp; for superoxide dismutase (SOD), forward primer was 5′-ATCCTCTTGGAACCCACCACA-3′ and reverse primer was 5′-ATCTGGACGCACATCTTTGT-3′, with a product size of 95 bp; for glutathione S-transferase 4A (GST4A), forward primer was 5′-AGACACATCGCCAGGACTAA-3′ and reverse primer was 5′-ACGGATACACGACTCCAACT-3′, yielding a product of 127 bp; finally, for the housekeeping gene 18S ribosomal RNA, forward primer was 5′-GATGGTGACTCTTGGATAACTTC-3′ and reverse primer was 5′-CTGCCTTCCTTGGATGTG-3′, producing a 189 bp amplicon.

After an initial denaturation step, the qPCR reaction was conducted with the following parameters: 95°C for 10 s, 60°C for 5 s, and 72°C for 10 s, repeated 39 times. Relative gene expression was calculated using the 2^−ΔΔCt method, with 18S rRNA as the housekeeping gene [24].

### Statistical analysis

All experiments were performed in triplicate, and results are presented as means and SD. Blood vessel counts were performed on five epoxy resin-embedded slides stained with crystal violet and basic fuchsin, analysing random 45 000 μm² fields using ImageJ (https://imagej.net/ij/index.html). Fluorescence intensities were also quantified with ImageJ.

Statistical analyses were performed with Prism 8.0 (GraphPad Software, La Jolla, CA, USA), using one-way ANalysis Of VAriance (ANOVA) with Dunnett’s post hoc test (*P* < .05). Relative mRNA expression was analysed by comparing ΔΔCt values of genes of interest to those of 18S, with significant differences indicated by asterisks in the graphs between N.T. and MPs/NPs-exposed leeches at each time point.

## Results

To aid navigation, the results are organized by biological domain: angiogenesis (CD31^+^), inflammation (*Hm*AIF-1), phagocytosis (ACP^+^ counts), and oxidative-stress responses (SOD, GST). For each endpoint, the time course (24 , 72 h, and 1 week) was reported versus N.T. Summary statistics are presented in Table 1 and representative image and graph outputs are shown in the figures.

**Table 1:** Angiogenic, inflammatory, phagocytic, and oxidative-stress readouts in *H. verbana* after exposure to MPs/NPs released from 3D-printed aligners at 24 , 72 h, and 1-week.

	N.T.	24 h	72 h	1-week
	Mean ± SD	Mean ± SD	Mean ± SD	Mean ± SD
CD31+	32 132 ± 2245	40 783 ± 1599 **	44 783 ± 2046 ***	43 102 ± 1973 **
HmAIF-1	1 ± 0.06	2.87 ± 0.22***	1.44 ± 0.12**	3 ± 0.01***
ACP+	5.29 ± 1.38	10.71 ± 1.98***	9 ± 2.38**	10.86 ± 2.48***
SOD	1 ± 0.04	2.63 ± 0.24***	1.34 ± 0.08	1.33 ± 0.07
GST	1 ± 0.03	3.16 ± 0.45***	1.45 ± 0.15*	0.82 ± 0.01

CD31+ denotes total endothelial immunofluorescence intensity; HmAIF-1 denotes macrophage-marker expression (relative immunofluorescence); ACP+ denotes counts of ACP-positive phagocytes; SOD and GST denote antioxidant gene expression by qPCR. Statistics: one-way ANOVA with Dunnett’s post hoc versus N.T.; *P < .05; **P < .01; ***P < .001.

### Morphological analyses of *H. verbana* tissues after microplastics and nanoplastics exposure

**Figure 1:**
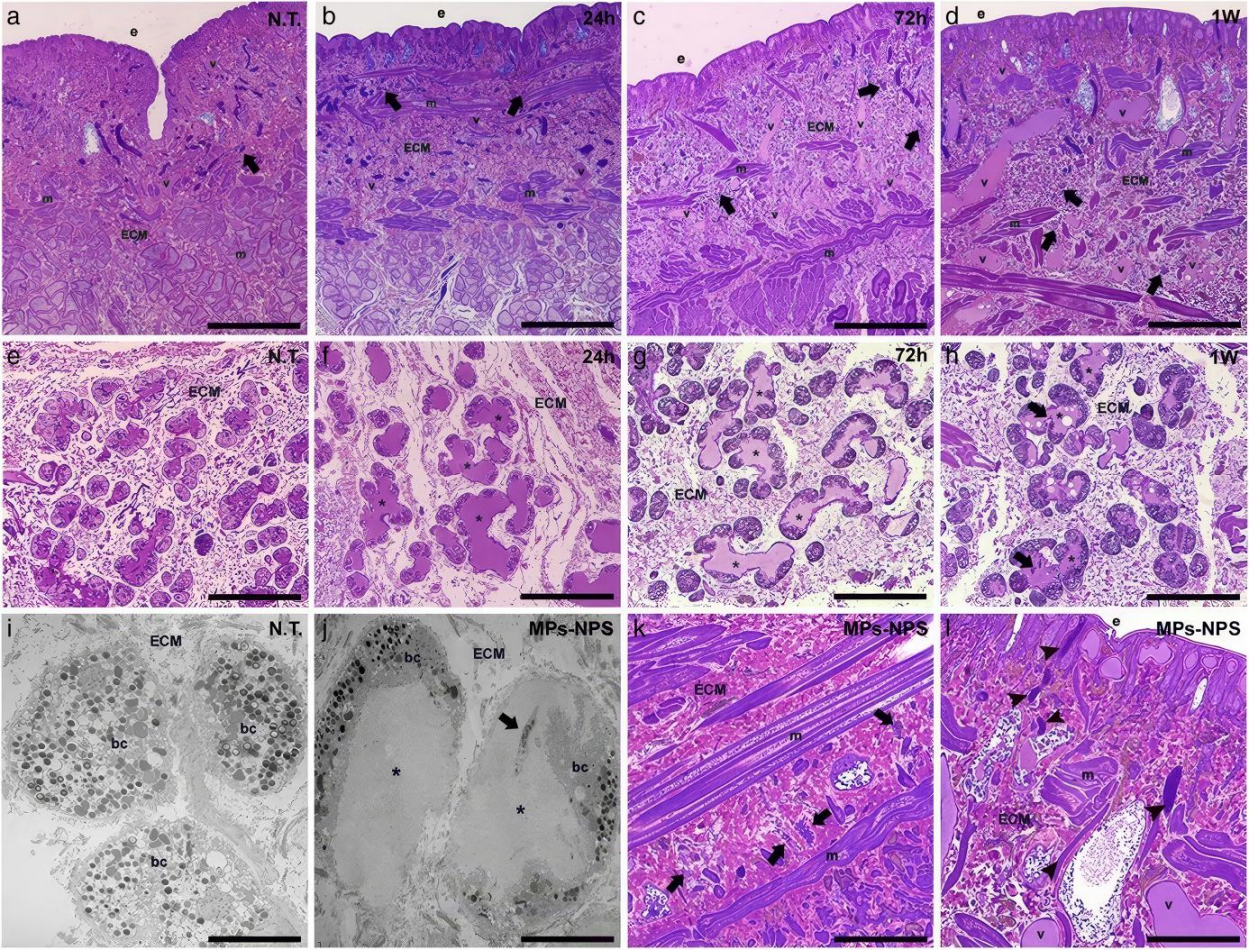
Morphological analyses of untreated (N.T.) and plastic-treated leeches. In control samples (a), tissues appeared predominantly avascular, with few immune cells (arrows) located beneath the epithelium (e) and surrounding muscle fibres (m) within the inner extracellular matrix (ECM). Exposure to plastic particles (b-d) induces the formation of new blood vessels (v) and the recruitment of immune cells (arrows). These findings are also confirmed by the activation of botryoidal tissue, whose conformation switches from a close cordon of cells (e, i) to a tubular structure (f–h, j), resulting from the distancing of botryoidal cells (bc), in which new vessel lumens become visible (asterisks). Circulating immune precursor cells (arrows in h, j) originate from botryoidal tissues and differentiate into mature macrophage-like cells (arrows in k). Numerous activated type II mucus cells (arrowheads) are visible following plastic freshwater administration (i). Bars in a–d: 100 µm; bars in e–h, j–l: 50 µm; bars in i, j: 10 µm.

To investigate the effects of MPs/NPs on the innate immunity of *H. verbana*, morphological analyses were performed using light and transmission electron microscopy (TEM) microscopy at 24, 72 h, and 1 week after exposure (Fig. 1). In nontreated animals, tissues appeared largely avascular, with few resident immune cells beneath the epithelium and around muscle fibres, and only occasional blood vessels and macrophage-like cells displaying cytoplasmic pseudopodia (Fig. 1a). In contrast, after 24 h of MPs/NPs exposure, blood vessel formation increased and continued to rise to 1 week (Figs. 1b–d), reflecting angiogenesis as a key stress response in leeches.

Botryoidal tissue activation, crucial for angiogenesis and haematopoiesis, was also assessed. In controls, this tissue formed a compact cordon (Fig. 1e), whereas exposure to MPs/NPs caused botryoidal cells to separate, forming new vascular lumens (Figs. 1f–h). Numerous haematopoietic precursor cells were also observed, particularly at 1 week (Fig. 1h), migrating through the new vessels towards plastic-affected areas. TEM analyses confirmed these findings, showing the formation of vascular structures and immune precursors (Figs 1i and j).

Alongside angiogenesis, marked recruitment of macrophage-like cells and activation of type II mucus cells were observed (Figs 1k and 1l). Macrophage-like cells, derived from immune precursors, exhibited numerous cytoplasmic pseudopodia, while activated mucus cells displayed an elongated morphology, suggesting that plastic particles elicit an inflammatory response in leech tissues.

### Evaluation of angiogenic induction following acute microplastics and nanoplastics exposure

**Figure 2:**
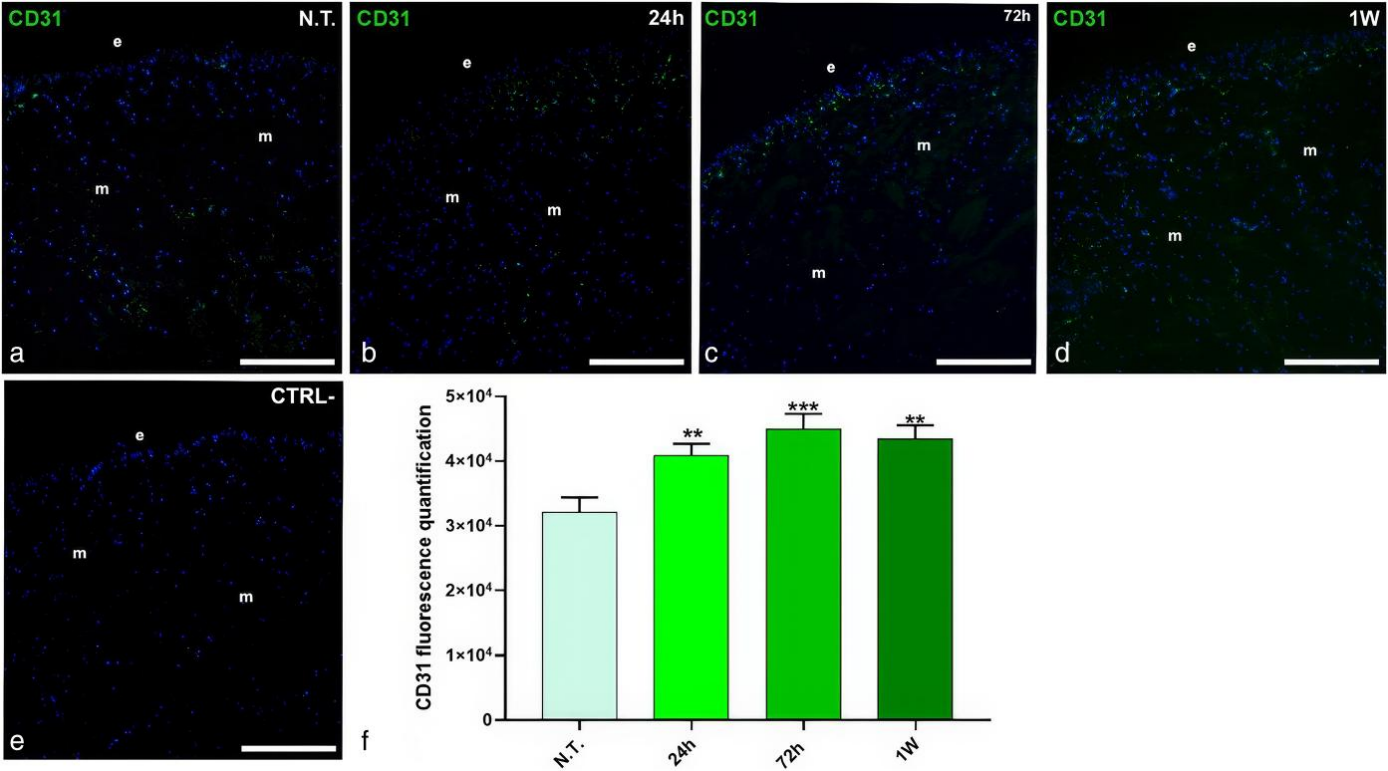
Immunofluorescence analyses of untreated (N.T.) (a) and plastic-treated leeches in the presence of anti-CD31 antibody. In control samples, a weak CD31 signal is visible beneath the epithelium (e) and around muscle fibres (m). At the same time, an increasing number of CD31+ cells were evident in the other time points analysed (b–d). In the negative control experiments (e), where the primary antibody was omitted, no signals were detected apart from the cell-nucleus-specific DAPI signal. Graph showing total CD31 fluorescence intensity (f). ** means *P* < .01; *** means *P* < .001. Bars in a–e: 100 µm.

To confirm the morphological findings, immunofluorescence experiments were performed on untreated and plastic-treated leeches using an anti-CD31 antibody, a specific endothelial cell marker (Fig. 2). In untreated animals, the CD31 signal was low, with only a few CD31^+^ cells beneath the epithelium and around muscle fibres, confirming the predominantly avascular tissue structure (Fig. 2a). In contrast, leeches exposed to MPs/NPs showed a marked increase in CD31 signal (Figs 2b–d). After 24 h, fluorescence intensity was already higher than in the controls (Fig. 2b), with further angiogenic activity observed at 72 h and 1 week (Figs. 2c and d). No fluorescence was detected in negative controls where the primary antibody was omitted (Fig. 2e). Quantification of CD31 fluorescence confirmed a significant increase in all plastic-treated animals (Fig. 2f). One-way ANOVA showed statistically significant changes in N.T. and exposed leeches over time (24 h, *P* < .01; 72 h, *P* < .001; 1 week, *P* < .01).

### Evaluation of macrophage-like immune cell activation

**Figure 3:**
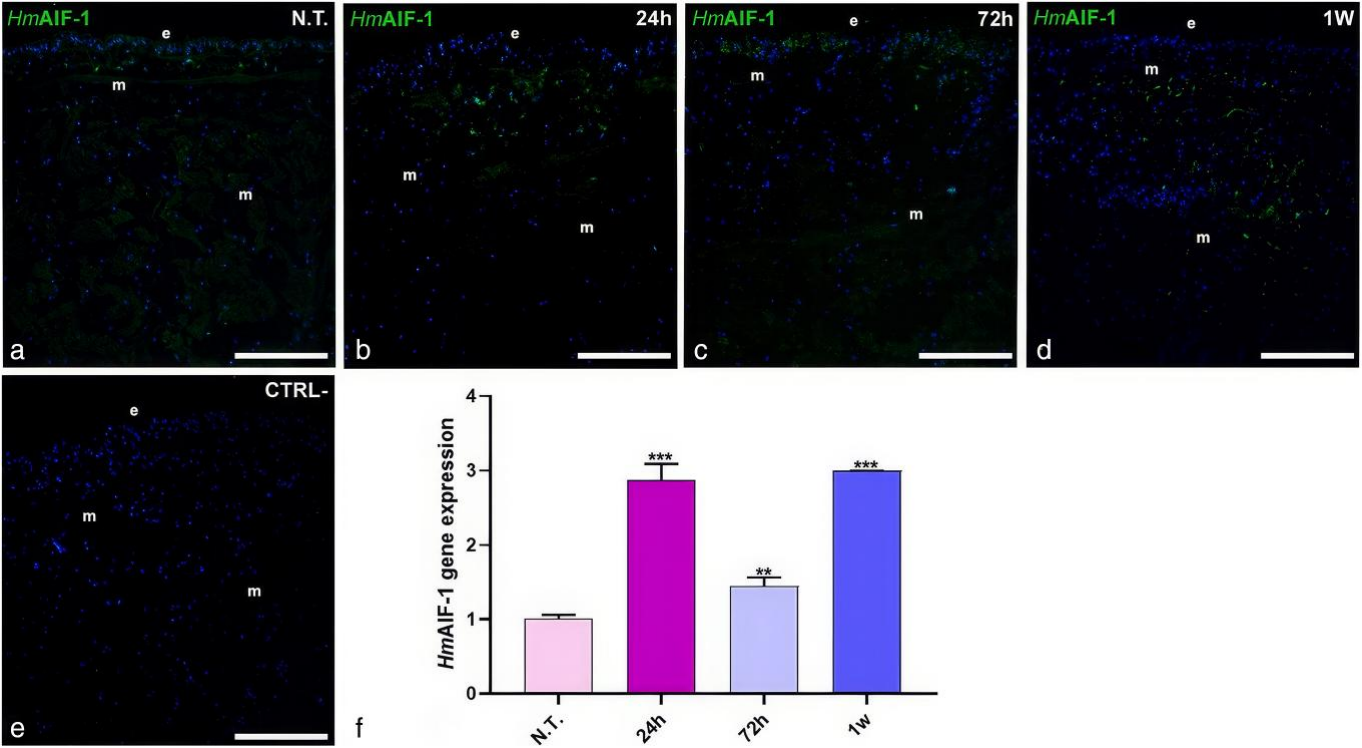
Immunofluorescence analyses of untreated (N.T.) (a) and plastic-treated leeches in the presence of anti-*Hm*AIF-1 antibody. In control samples, a weak signal for *Hm*AIF-1 is visible underneath the epithelium (e) and surrounding muscle fibres (m). At the same time, an increasing number of *Hm*AIF-1+ cells were evident in the other time points analysed (b–d). In negative control experiments, in which the primary antibody was omitted, no signals were detected other than the cell-nucleus-specific DAPI signal (e). *Hm*AIF-1 qPCR analyses (f). The 18S ribosomal RNA gene is used as a housekeeping reference gene. ** means *P* < .01; *** means *P* < .001. Bars in a–e: 100 µm.

The expression of *Hm*AIF-1, a pro-inflammatory cytokine specific to leech macrophages, was assessed by immunofluorescence and qPCR (Fig. 3). Immunological analysis revealed a significant increase in *Hm*AIF-1 expression in plastic-treated animals compared to controls (Fig. 3a), particularly at 24 h and 1 week (Figs. 3b and d), with numerous *Hm*AIF-1-positive cells beneath the epithelium. At 72 h post-exposure, HmAIF-1 expression remained significantly higher than in control animals. However, it showed a slight decrease in signal intensity compared to levels at 24 h and 1 week, suggesting a transient modulation of the inflammatory response (Fig. 3c). No signal was detected in negative controls lacking the primary antibody (Fig. 3e). qPCR confirmed these findings, showing significant expression peaks at 24 h and 1 week compared to controls (Fig. 3f). Statistical analysis revealed a significant difference between N.T. and treated animal samples at any time point (24 h, *P* < .001; 72 h, *P* < .01; 1 week, *P* < .001).

### Acid phosphatase histoenzymatic assays

**Figure 4:**
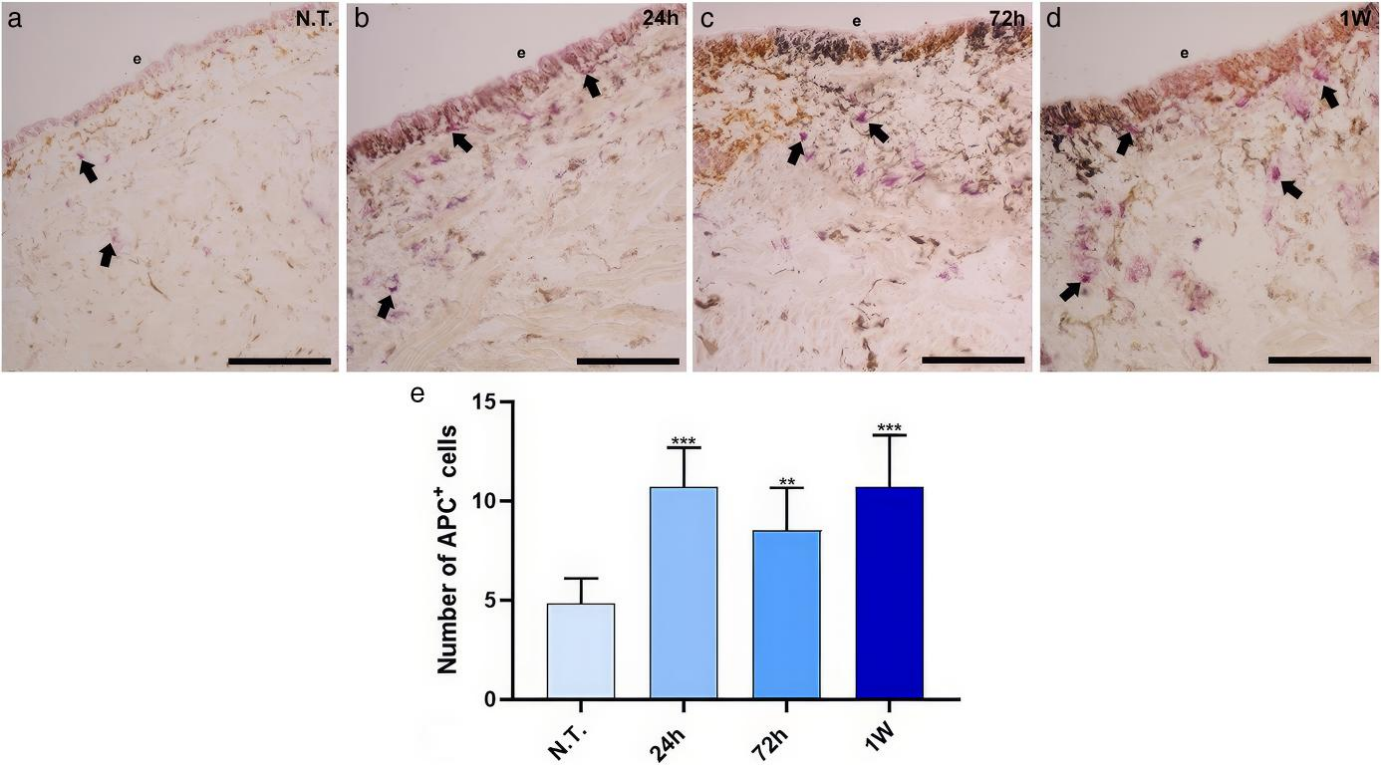
ACP assay for analysing the phagocytic cells recruitment evaluation following plastic particle exposure. Few positive cells are visible in control samples (a), compared with treated leeches (b–d), in which the total number of ACP-positive cells increases, as shown in graph (e). ** means *P* < .01; *** means *P* < .001. Bars in a–d: 100 µm. e: epithelium; arrows: ACP-positive macrophages.

In medicinal leeches, debris and exogenous particles are removed by macrophage-like cells through phagocytosis and encapsulation. To confirm their role in clearing MPs and NPs, a histoenzymatic ACP assay was performed to detect active phagocytes (Fig. 4). In control samples (Fig. 4a), only a few ACP^+^ resident macrophages were observed. After 24 h of MPs/NPs exposure, their number markedly increased, particularly near the epithelium (Fig. 4b). Although a slight reduction was noted at 72 h (Fig. 4c), the number of ACP + cells remained elevated after 1 week (Fig. 4d) compared to N.T. controls. These results were confirmed by quantifying the total number of ACP^+^ cells (Fig. 4e). Statistically, the number of ACP^+^ cells was significantly increased in the samples exposed to MPs/NPs compared to the unexposed ones (24 h, *P* < .001; 72 h, *P* < .01; 1 week, *P* < .001).

### Assessment of the oxidative stress induced by qPCR

**Figure 5:**
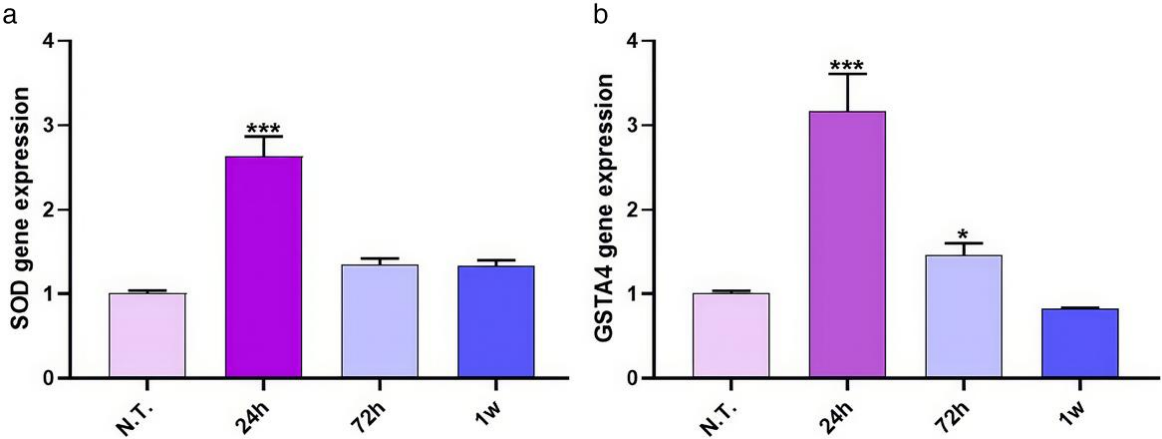
SOD and GSTA4 antioxidant enzymes qPCR analyses (a and b). The graphs show the expression of both genes in control tissues (N.T.) or plastic-treated samples. The 18S ribosomal RNA gene is used as a housekeeping reference gene. * means *P* < .05; *** means *P* < .001. One-way ANOVA revealed a significant effect of treatment on SOD expression: *F*(3, 8) = 88.5, *P* < .001. Similarly, treatment had a significant effect on GST expression: *F*(3, 8) = 59.78, *P* < .001.

Given that MPs/NPs of plastics usually induce oxidative stress in living organisms, the production of reactive oxygen species (ROS) and the resulting cellular damage have been evaluated. The gene expression of two specific antioxidant enzymes, superoxide dismutase (*SOD*) and glutathione transferase (*GST*), has been assessed. In detail, qPCR analyses revealed that, compared to control samples, the presence of plastics significantly induced the activation of these specific enzymes after 24 h from exposure (*P* < .001), as shown in the relative graphs (Fig. 5a and b). Conversely, at 72 h and 1 week, SOD and GST expression appeared reduced, with significant alteration only for GST (*P* < .05), which returned to basal levels by 1 week (*P* > .05).

## Discussion

The growing use of 3D-printed aligners has raised concerns about their biocompatibility. Since patients wear aligners daily for extended periods, continuous exposure to plastic particles may have a negative impact on local and systemic health through the progressive release and cumulative effects. Although the impact of MPs/NPs on biological systems has been widely studied [27, 28] Limited data exist for orthodontic materials. This study demonstrates for the first time that even short-term exposure to plastic particles triggers angiogenesis, immune activation, and oxidative stress, supporting their potential harmful effects. These findings align with previous evidence of the cytotoxic and immunomodulatory activity of plastic-derived particles in biological and *in vitro* models [16, 29].

This histological and immunofluorescence study showed a marked increase in blood vessel formation in leeches exposed to MPs/NPs from 3D-printable resins, suggesting that plastic particles stimulate angiogenic pathways. The upregulation of CD31 confirms this angiogenic response, a physiological mechanism linked to tissue repair and stress adaptation [23]. These findings support the hypothesis that MPs/NPs act as pro-inflammatory agents, activating innate immunity and potentially leading to chronic inflammation [30].

Structural changes in botryoidal tissues, shifting from compact cords to dispersed formations with vascular lumens, further indicate disruption of tissue homeostasis. A strong immune response was also observed, with activation of macrophage-like and type II mucus cells, consistent with prior studies reporting immune cell recruitment following plastic exposure [31]. The increased expression of *Hm*AIF-1, a macrophage-specific cytokine, supports this activation, similarly to vertebrate models [21]. Enhanced ACP-positive cells activity further suggests increased phagocytosis, confirming that immune cells attempt to clear plastic particles from tissues.

Another key concern is the induction of oxidative stress by plastic exposure, leading to cellular damage [32]. qPCR analysis showed a significant increase in *SOD* and *GST* gene expression at 24 hours, indicating an early oxidative-stress response. Expression levels returned to baseline by 72 hours and 1 week, suggesting a transient but intense oxidative burst. This aligns with studies in other invertebrate and vertebrate models, where NPs triggered oxidative stress, DNA damage, mitochondrial dysfunction, and lipid peroxidation [33, 34]. Such plastic-derived particles significantly increased oxidative stress and DNA damage *in vitro*, with similar effects in reproductive tissues. As oxidative stress drives inflammation and apoptosis, these results suggest that exposure to MPs/NPs may have lasting effects on tissue function and integrity, warranting further investigation.

Although recently introduced in orthodontics, 3D-printable resins have been widely studied *in vitro* and have been shown to exhibit higher cytotoxicity and genotoxicity than thermoformed devices [35]. Unlike thermoformed aligners, which degrade mainly through mechanical wear, 3D-printed aligners face additional chemical degradation risks due to their photopolymerized composition. Intraoral friction, temperature changes, and enzymatic activity may accelerate polymer breakdown [36]. Moreover, residual monomers not fully polymerized during post-curing, influenced by resin thickness, may increase cytotoxicity [37, 38]. These uncured components, trapped between printed layers, could gradually release as MPs/NPs or detach mechanically. Temperature fluctuations and enzymatic degradation from saliva may further exacerbate this process [39]. Notably, some monomers, such as urethane dimethacrylate, have been linked to cytotoxic, genotoxic, and oestrogenic effects, raising concerns about chronic exposure in the oral cavity [36, 40]. Their release could intensify inflammatory responses in oral tissues and in animal bioassays, thereby amplifying histological and molecular changes induced by plastics alone. However, the inability to chemically characterize these resins remains a limitation in identifying specific monomers in human fluids and tissues.

This study also has some limitations. First, *H. verbana* may not fully reflect the complexity of human oral tissues, particularly in epithelial turnover and immune response. Additionally, the exposure conditions do not replicate the intraoral environment, where saliva, pH, and microbiota influence particle release and degradation. Further studies using mammalian models or human tissue cultures are needed. Another limitation is the absence of direct quantification of MPs/NPs during real aligner wear, as well as limited data on their accumulation and systemic effects. Moreover, as only one resin type was tested and its exact chemical composition is unknown, further research comparing different materials is required to generalize these findings. Future works should also characterize the released particles and assess their distribution and potential systemic impacts. Lastly, the MPs/NPs dosage used here may underestimate clinical exposure levels.

The present data indicate that acute tissue responses are sensitive to particle burden and to the potential presence of residual reactive species from photopolymer resins, including those in eluents, as recently demonstrated [41]. Consequently, modifiable manufacturing levers include resin chemistry, the final degree of conversion after polymerization, and postprocessing parameters. Therefore, from a clinical standpoint, adopting postprocessing protocols that maximize conversion and minimize extractables, plus quality checks for particle release under cyclic thermo-mechanical loading, may help reduce MPs/NPs exposure.

For future research, long-term safety requires models that quantify time-resolved MP/NP release under cyclic thermo-mechanical loading and saliva exposure, compare multiple aligner resins and postprocessing protocols, and validate these findings across mammalian mucosal tissues and co-culture systems with longitudinal endpoints. Prior *in vitro studies* demonstrated resin-dependent cytotoxicity and leachate profiles [36–38, 41]; integrating these data with dynamic, clinically oriented bioassays such as the present one provides a translational framework to prioritize materials and optimize postprocessing, thereby mitigating health risks.

## Conclusions

This study demonstrated that MPs and NPs released from 3D-printed aligners trigger angiogenic, inflammatory, and oxidative-stress responses in the *H. verbana* model, raising concerns about their potential oral and systemic effects. Given the increasing use of 3D-printed aligners, future research should investigate long-term exposure in mammalian models and explore mitigation strategies, such as the use of improved materials or coatings to limit particle release.

However, this work is not a critique of 3D-printed resins, which remain highly promising, but highlights the limitations of current biocompatibility tests. These protocols, which are mostly static, do not accurately reflect the dynamic release of MPs/NPs during clinical use. Future studies should utilize models comparable to human systems (such as vertebrate or murine models) to better capture particle release and long-term biological responses.

## Data Availability

The data generated and/or analysed during the current study are available from the corresponding author on reasonable request.
